# Exercise and Nutrition Impact on Osteoporosis and Sarcopenia—The Incidence of Osteosarcopenia: A Narrative Review

**DOI:** 10.3390/nu13124499

**Published:** 2021-12-16

**Authors:** Sousana K. Papadopoulou, Konstantinos Papadimitriou, Gavriela Voulgaridou, Evridiki Georgaki, Eudoxia Tsotidou, Olga Zantidou, Dimitrios Papandreou

**Affiliations:** 1 Department of Nutritional Sciences and Dietetics, School of Health Sciences, International Hellenic University, 57001 Thessaloniki, Greece; sousana@the.ihu.gr (S.K.P.); gabivoulg@gmail.com (G.V.); tsotidis.family@gmail.com (E.T.); olgakizantidou1995@gmail.com (O.Z.); 2 Laboratory of Biological Evaluation of Human Performance, School of Physical Education and Sport Science, Aristotle University of Thessaloniki, 57001 Thessaloniki, Greece; kostakispapadim@gmail.com; 3 Barts and the London School of Medicine and Dentistry, Queen Mary University of London, London E1 4NS, UK; evridiki.georgaki@outlook.com; 4 Department of Health, College of Natural and Health Sciences, Zayed University, Abu Dhabi 144534, United Arab Emirates

**Keywords:** osteosarcopenia, osteoporosis, exercise, vitamin D, calcium

## Abstract

Osteoporosis and sarcopenia are diseases which affect the myoskeletal system and often occur in older adults. They are characterized by low bone density and loss of muscle mass and strength, factors which reduce the quality of life and mobility. Recently, apart from pharmaceutical interventions, many studies have focused on non-pharmaceutical approaches for the prevention of osteoporosis and sarcopenia with exercise and nutrition to being the most important and well studied of those. The purpose of the current narrative review is to describe the role of exercise and nutrition on prevention of osteoporosis and sarcopenia in older adults and to define the incidence of osteosarcopenia. Most of the publications which were included in this review show that resistance and endurance exercises prevent the development of osteoporosis and sarcopenia. Furthermore, protein and vitamin D intake, as well as a healthy diet, present a protective role against the development of the above bone diseases. However, current scientific data are not sufficient for reaching solid conclusions. Although the roles of exercise and nutrition on osteoporosis and sarcopenia seem to have been largely evaluated in literature over the recent years, most of the studies which have been conducted present high heterogeneity and small sample sizes. Therefore, they cannot reach final conclusions. In addition, osteosarcopenia seems to be caused by the effects of osteoporosis and sarcopenia on elderly. Larger meta-analyses and randomized controlled trials are needed designed based on strict inclusion criteria, in order to describe the exact role of exercise and nutrition on osteoporosis and sarcopenia.

## 1. Introduction

### 1.1. Definition and Demographic Data of Osteoporosis and Sarcopenia

#### 1.1.1. Osteoporosis

Osteoporosis is a silent disease, without any clear clinical symptoms, until a fracture occurs. Fractures are a major public health burden, as they are the main causes of morbidity, impairment, decreased quality of life and mortality [1]. Osteoporosis has a lot of medical, economic and social consequences. The total burden of osteoporosis is estimated to grow by 50% with more than 3 million incident fractures by 2025, a cost that is translated into almost USD 25.3 billion per year in the US [2]. 

Worldwide, 200 million people suffer from osteoporosis and 8.9 million fractures occur every year [3]. By 2050, hip fractures may exceed 21 million cases [1]. The prevalence of osteoporosis is 18.3% globally, and it is greater in women than in men (23.1 and 11.7, respectively) [4]. The direct cost of treating these osteoporotic fractures in five European countries (France, Germany, the United Kingdom, Italy and Spain) is EUR 29 billion, while for the 27 EU Member States as a whole it is EUR 38.7 billion, a cost that is expected to increase by 25% until 2025 [5]. 

#### 1.1.2. Sarcopenia

Another additive syndrome which also affects humans is sarcopenia. Sarcopenia is not a syndrome which affects only daily life, reducing quality of life and strength, increasing likelihood of falls and loss of autonomy [6], but also leads to osteoporosis, obesity and impairs metabolic health [7]. It has been shown that the loss of muscle mass that accompanies sarcopenia leads to increased insulin resistance, which promotes the development of metabolic syndrome and obesity [8]. 

The origin of sarcopenia is multifactorial and its clinical significance, although universally recognized, it is not universally accepted [9]. According to the European Working Group of Sarcopenia in Older People (EWGSOP2), sarcopenia requires the presence of both low muscle mass and low muscle function. This group defines sarcopenia as an age-related syndrome characterized by a progressive and generalized loss of skeletal muscle mass and strength with adverse effects on human health.

A concomitant increase in fat mass may also be present. The EWGSOP2 set two standard deviations below a healthy population the recommended cut-off points for muscle mass. Cut-off values of gait speed and handgrip strength were <0.8 m/s and <30 kg in men and <20 kg in women, respectively [10]. SDOC suggested sex-specific cut-off points for muscle weakness (low handgrip strength) and slowness (slow gait speed) and depict higher values for muscle strength cut-off points, while they do not take into account muscle mass (<35.5 for men and <20 for women and 0.8 m/s for gait speed) [11]. 

The prevalence of sarcopenia varies between different populations, with reported rates of 5–50% in people over 65 years of age [12]. These variations depend on factors such as the specific techniques used to measure muscle mass and muscle function, the population under study and diagnostic criteria [12]. Sarcopenia appears in 5–13% in the seventh decade of life and can increase up to 11–50% by the age of 80 years. Furthermore, it is predicted that it will affect more than 500 million elderly people by 2050 [13].

Over time, the lifestyle factors of physical activity and nutrition contribute to the downregulation of many syndromes and disease symptoms. Thus, the purpose of the present narrative review is to underline the effects of physical exercise and nutrition on osteoporosis and sarcopenia and the incidence of osteosarcopenia. 

## 2. Methodology 

### 2.1. Narrative Review Construction

The present narrative review was organized through the “Narrative Review Checklist” which is proposed by the Academy of Nutrition and Dietetics. Thus, we carried out specific checks of manuscripts’ structures and a careful selection of the articles which are included in the manuscript [14]. 

### 2.2. Studies Selection

The search was carried out in three electronic databases PubMed, Scopus and Google Scholar. The search strategy included studies published from January 2015 until now. However, some included review studies contained results from high-quality studies which in some cases date before 2005. The pre-defined search terms were: “osteoporosis” or “sarcopenia” or “osteosarcopenia” and “bone mass loss” or “muscle mass loss” or “bone mass density” or “muscle strength” and “physical exercise/performance” or “physical function” and “nutrition”. For a more targeted and comprehensive search, the above words were combined with other, more specific terms such as “vitamins” or “supplements/dietary” or “resistance training/resistance exercise training” or “aerobic exercise” or “endurance training/endurance exercise training” or “proteins and hormones”. 

All published studies retrieved from the literature databases were collected and sorted by date and type of intervention in order to reduce heterogeneity, and then double entries were removed. This review included both observational cohort, case-control, cross-sectional studies, systematic reviews and meta-analyses, and randomized, double-blind studies (randomized controlled trials, RCTs).

Animal and experimental model studies were excluded from the review. In addition, studies with a small sample size were excluded, as well as studies which did not adequately specify the selection criteria or included groups of subjects receiving medication for another disease that affected bone or muscle metabolism. Finally, case reports, editorials, letters to the editor and conference proceedings were excluded from the review. A total of 100 references were included in this review.

## 3. Discussion/Summary

### 3.1. Osteoporosis

#### Mechanism

Osteoporosis is mostly caused by an imbalance between the action of osteoblasts and osteoclasts. The three main components which are affected in osteoporosis are osteoblasts, osteocytes and osteoclasts [15]. However, the decrease in estrogen seems to be a prevalent mechanism in osteoporosis, notably in menopause. The reduction in estrogen production causes a sequence of alterations on the T-cells and the subclass T regulatory cell (T-reg). 

Thus, it is observed to be associated with an increase in pro-inflammatory cytokines (IL-1, IL-6 IL-17, TNF-α) secretion, which inhibit osteoclast regeneration. Moreover, a similar mechanism was found for B-cells whose upregulation is correlated with osteoporosis. Furthermore, gut microbiomes (GM) correlated with osteoporosis. The absorption of nutrients via intestine is vital for human tissues regeneration. Therefore, the poor nutrition or prebiotic diets may affect the GM’s secretion, causing osteoporosis. The last possible factor is the senescence-associated secretory phenotype (SASP). According to the mechanism, the increase in senescent cells contributes to the appearance of osteoporosis [16].

### 3.2. Exercise and Nutrition Impact on Osteoporosis

#### 3.2.1. Exercise

Although there is heterogeneity between studies, most of them suggest that both weight-bearing and resistance exercises have the optimal effect on prevention and treatment of osteoporosis in older people [17,18,19,20,21]. However, according to the existing literature, it is concluded that these data are so far very limited and further research is needed in order to draw clear conclusions. 

A review study of Harding and Beck (2017) demonstrated that bone-targeted programs acted positively on bone mineral density (BMD) and bone mineral content (BMC) of loaded bones [22]. Exercise influences bone strength and mass at all ages. Thus, regular physical activity promotes bone mass increase and bone geometry optimization during childhood and puberty, contributes to bone mass maintenance during adulthood, and reduces the decrease in bone mass loss and strength during old age, preventing osteoporotic fractures in the elderly [23]. 

However, high intensity and high volume of training together with low energy availability can lead to menstrual dysfunction and decreased bone mineral density and delayed bone growth [24]. Thus, the contribution of nutrition on different types of exercise and especially on resistance exercise is vital for the increase of bone formation. 

The National Osteoporosis Foundation (NOF) suggested high or low impact weight-bearing and muscle-strengthening exercises to prevent osteoporosis [25]. These types of exercises include jumping, jogging and aerobics as high impact exercises as well as walking and step aerobics as lower impact exercises. In addition, muscle strengthening exercises include lifting weights, using elastic exercise bands and exercises including some resistance against gravity. Despite the benefits of walking on body composition and cardiometabolic health, it has marginal or no effects on the prevention of osteoporosis [26]. On the other hand, the LIFTMOR study shows that the combination of high-intensity progressive resistance and impact weight-bearing training has more benefits for BDM at the lumbar spine than a home-based low-intensity program in postmenopausal women [27]. On postmenopausal woman, long-term resistance or aerobic exercise contributes to the increase in bone formation and mass [28,29]. Specifically, an exercise program accompanied by weight-bearing and resistance activities tends to increase markers of bone formation, namely pro-collagen type 1 N-terminal peptide (P1NP) levels and osteogenic cells (OCs), whereas it little or no increase in the markers of bone resorption was observed [28]. Therefore, aerobic exercise is efficient in both attenuating bone resorption raise and enhancing the bone formation as well. Another type of exercise which has been investigated is aquatic exercise. Swimming generally is associated with little or no effect on BMD [30,31]. In addition, Moreira et al. investigated a high-intensity exercise program showing that it was efficient in bone formation increasing the formation marker P1NP and simultaneously limiting the increase of bone resorption marker [29]. Further research is necessary due to the lack of research regarding the role of aquatic exercise and its effects on BDM. Additionally, a multidimensional strength training program that stimulates daily activities has the best effect on improving activities that require fast and explosive muscle contractions, fast reaction, muscle coordination and balance [32].

#### 3.2.2. Nutrition

The consumption of milk and dairy products reduces the risk of osteoporosis. Malmir et al. (2020) [33] in a meta-analysis study confirmed that dairy consumption is not associated with prevention against osteoporosis and fractures. However, protein and vitamin D supplementation in older people seems to prevent osteoporosis and fractures by increasing bone density [34]. On the other hand, RCTs studies do not confirm a reduction in the incidence of falls and fracture after vitamin D supplementation [35]. The PROVIDE study with 380 sarcopenic older adults depicts the important role of leucine and vitamin D. More specifically, a group with both higher baseline values for vitamin D 25(OH)D concentration and protein intake had the best outcome in muscle gain [36]. 

Although the importance of proper nutrition in older people has long been recognized, research evaluating the effects of dietary habits on muscle and bone mass is relatively recent. As age increases, there is a decline in energy intake, which can reach as high as 16–20% in elders >65 years [37]. Older people may eat more slowly, eat fewer and smaller meals and have a reduced appetite [38]. However, in addition to reduced food intake, the quality of the diet also plays an important role in muscle strength in elders [39]. Dietary patterns such as the Mediterranean diet, i.e., rich in vegetables, fruits, fish and good fats, enhance muscle strength and functionality in older people [39]. 

A recent meta-analysis of Tai et al. (2015) [40] showed that BMD of the lumbar spine, total hip, femoral neck, total body was slightly increased (up to 1.85) by increasing dietary sources of calcium or taking calcium supplements. However, BMD increases were small and non-progressive without providing any reduction in BMD loss rates over one year. These results suggest that BMD was not beneficially affected by the non-calcium dietary components. Similarly, a meta-analysis of Reid et al. (2014) [41] showed that vitamin D monotherapy did not affect BMD and thus was inappropriate for preventing osteoporosis in a population without vitamin D deficiency. 

In contrast, another study contributed to research on the anti-osteoporotic properties of vitamin K2, showing that MK-7 supplements can prevent bone loss at the lumbar spine and femoral neck in postmenopausal women and have a positive effect on bone strength [42]. Several micronutrients seemed to be implicated in bone metabolism. Thus, except for calcium, vitamin D and vitamin K, zinc, copper, magnesium and manganese were presumed to be important for osteoporosis prevention, while intake of fluoride and strontium seem to be of critical importance in stimulating osteoblasts and inhibiting osteoclasts [43]. A recent literature review indicates that high protein intake may have a protective role in bone density at the lumbar spine, compared to low protein intake in adults [44]. Furthermore, adequate intake of fruits and vegetables seems to have a positive effect on bone density [45] (Table 1).

### 3.3. Sarcopenia

#### Mechanism

Sarcopenia is a multifactorial syndrome; thus, the explanation of its cause is still under study, although it has been correlated with the appearance of many symptoms. Inflammation is one of them through the secretion of interleukins (IL-1, IL-6), CRP and tumor necrosis factor–α (TNF–α) [48]. The inflammatory response induces a reduction on satellite cells production causing degradation of muscle tissue [48]. Furthermore, inflammatory upregulation in relation with the increase of E3 ubiquitin and MuRF–1 provokes the decrease of the ubiquitin–proteasome system (UPS) which is connected to the degradation of muscle tissue [48]. 

Another metabolic path through which inflammatory response is upregulated and satellite cells are decreased is through the increase of p38 MAPKs which upregulate the p16Iu4a [49]. Satellite cells seem to play a central part in sarcopenia; this can be enhanced due to the fibroblast growth factors (FGFs) mechanism. The increase of FGF2 and decrease of FGF6 induces a downregulation of satellite cells, again causing muscle tissue degradation [50]. Reactive oxygen species (ROS) is another central mechanism in which its upregulation negatively affects the mitochondrial function, causing myosteatosis, a state where adipose tissue depots in skeletal muscle [51]. Furthermore, mitochondrial downregulation is connected with the increase of the autophagy [52], a catabolic process which has been found to cause sarcopenia [48]. 

### 3.4. Exercise and Nutrition Impact on Sarcopenia

#### 3.4.1. Exercise

The majority of studies found that exercise improves muscle mass, strength and function, so may have a protective and beneficial role against sarcopenia, through the increase in muscle mass and strength and mobility improvement while less active individuals have an increased risk for developing sarcopenia or increasing its severity [53,54,55,56,57,58,59].

Both muscle size and architecture change with advancing adulthood. A previous study reported a reduction in muscle mass followed by a 30% to 40% decline in the number of muscle fibers between the second and eighth decade [60]. The size of muscle fibers is also affected, but to a lower degree. Type II muscle fibers are 10–40% smaller in older than in younger people [61], whereas type I muscle fiber size is largely unaffected [48]. On the other hand, the increase of the cross-sectional area of type I and II muscle fibers, and lean body mass in elderly individuals, leads to the increase in muscle strength [62]. 

A multidimensional strength training program that simulates daily activities has the best effect on improving daily activities requiring fast and explosive muscle contractions, fast reaction, muscle coordination and balance [32]. It is well known that resistance training enhances cross-sectional area and size of muscle fibers, particularly types IIa and IIx (fast-twist fibers) rather than type I [63]. Beckwee et al. suggested a high intensity resistance training program in order to achieve maximum strength gains, while a low intensity resistance training program is adequate to cause an increase in strength [53]. On the other hand, aerobic exercise training increases mitochondrial biogenesis [64] and could enhance muscle hypertrophy and strength [65]. Furthermore, moderate load eccentric exercises have been shown to be as effective as conventional strength training in increasing muscle volume and strength [66] and consequently reducing the risk of falls and improving both mobility and quality of life [67]. 

Last but not least, balance exercises and specifically postural types of training on unstable and stable surfaces seem to contribute to the improvement of body balance [68]. Interventions which last more than 8 weeks and include static balance training and the strengthening of lower limbs act beneficially on the improvement of dynamic balance, resulting in a greater stability. This improvement positively affects walking ability and walking speed but reduces the single leg stance phase [68]. 

Balance ability is in accordance with the types of muscle fibers. Thus, a high percentage of type II fibers contribute to a fast reaction but a quick fatigue, whereas a high percentage of type I fibers are effective for standing abilities without early fatigue. So, this statement demonstrates that instability in elderly populations is caused because of frailty and muscle mass degradation, factors which are affected due to the switch of muscle fibers from I to II. Therefore, the importance of the type of exercises for sarcopenia syndrome is vital for patients [69]. 

According to various studies reviewed by Marty et al. (2017), the types of physical activities improve muscle mass, strength and function [70]. The combination of exercise and proper nutrition induces mitochondrial biogenesis and function and increases the number/function of satellite cells, while inhibits inflammatory cytokines, leading to increased protein synthesis and decreased protein degradation [71]. 

In addition, regular exercise can combat muscle dysfunction as well as neuromuscular damage caused by ageing. Non-mass-dependent muscle factors, such as muscle fiber length and tendon stiffness, were also increased by 10% and 64%, respectively, after exercise interventions in the elderly [72]. Various types of exercise can have a positive effect on an individual’s health, with resistance exercises having the best results [17,21].

#### 3.4.2. Nutrition

A balanced diet plays an important role in overall health and bone health, providing energy, macronutrients, vitamins and minerals. However, older people consume less energy and protein compared to younger people, even though their nutritional needs are often higher [73]. Both inadequate nutrient intake and physical inactivity increase the likelihood of falls and fractures or osteoporosis and sarcopenia [74]. Physical frailty as a result of a decline in multiple biological systems in functioning, together with stress factors, increases the risk of osteoporosis and sarcopenia [75]. 

Dietary patterns, such as the Mediterranean diet, probably have a positive effect on muscle maintenance, as it provides antioxidants that reduce oxidative stress, one of the main causes of sarcopenia [76,77]. Many RCTs studying the effect of protein or vitamin supplementation on muscle maintenance and the progression of sarcopenia confirm their essential role in preventing the disease. It is observed that especially proteins rich in leucine play an important role because they have anabolic properties [54,78,79]. Leucine supplementation leads to increases in protein synthesis rate, body mass and lean mass in the elderly [80]. HMB, a leucine metabolite, signals mTOR pathway leading to an increase in protein synthesis and simultaneously lower ubiquitin pathway, resulting in decreased protein degradation, while through muscle cholesterol provides an increased substrate for cell membrane repair [81]. 

ESPEN suggests a diet consisting of at least (a) 1.0–1.2 g protein/kg body weight/day for healthy elderly people, and (b) 1.2–1.5 g protein/kg body/day weight for elderly people with chronic or acute illness. However, many health professionals often express concern that protein-rich diets will overwhelm and exacerbate disturbed kidney function in the elderly. According to guidelines regarding the elderly with healthy kidney function or mild dysfunction, the aforementioned protein recommendation is safe. In patients with a moderately reduced glomerular filtration rate (GFR) or other forms of chronic kidney disease (CKD), health professionals should take into account the balance between risks of immobility due to falls and death and risk of developing final-stage kidney disease, while applying the clinical guidelines, in order to make the right decision. It is noticeable that patients diagnosed with severe CKD are usually recommended a lower amount of 0.6–0.8 protein/kg body weight/day [82].

Regarding vitamin D, there are many studies showing an association with sarcopenia. However, the level and frequency of dosage has not yet been clarified, nor the duration of treatment that may help improve muscle mass and function. Dietary interventions including whey protein, essential amino acids and vitamin D improve muscle mass and physical performance [83]. 

Although some studies have been carried out, the effectiveness of the combined action of exercise and diet in improving fitness and preventing disease in older people has not yet been established. The SPRINTT (Sarcopenia and Physical Frailty IN older people: multi-component Treatment) clinical trial is the largest and longest-running study designed to evaluate the effectiveness of complex non-drug therapeutic interventions to prevent motor difficulties in older sarcopenic patients [84]. It is to some extent a continuation of the concept of the LIFE study, which we have previously reported on, and will have a duration of 36 months trying to evaluate the effect of diet and exercise on patients’ physical activity [85] (Table 2).

### 3.5. Osteosarcopenia

Over the last six years, researchers have studied the pathophysiological mechanisms of a new term which is called osteosarcopenia or sarco-osteopenic, a condition in which both symptoms of osteoporosis and sarcopenia are observed [93]. Osteosarcopenia is a syndrome described by the co-existence of osteoporosis and sarcopenia, with the same clinical and biological features [94]. The relationship between osteoporosis and sarcopenia is reasonable in the context of the bone–muscle subunit. Both tissues are derived from a common mesenchymal projector stem cell [70]. Muscle cells secrete bone-regulated cytokines, while bone cells secrete IGF-1, which has potential muscle-stimulating properties [70]. Osteoporosis and sarcopenia are two conditions which share many similarities, including high prevalence, high socioeconomic costs, mechanisms of action and crucial effects on patients’ quality of life [9]. In addition, both lead to losses in bone mass and muscle quality, respectively, which are age-related but exacerbated by the presence of these diseases [9]. Furthermore, sarcopenic obesity, which is observed in elders, may increase the risk of cardiometabolic diseases, disability, and mortality and accelerate the decrease of physical function, because of synergistic complications from both sarcopenia and obesity [95]. Obesity, sarcopenia and osteoporosis may coexist as an entity called “osteosarcopenic obesity”, with patients experiencing health problems more severely than individuals with only one of these disorders [96] (Table 3). 

## 4. Conclusions

Osteoporosis and sarcopenia are major health problems that occur during ageing. Their prevention is particularly important as they are associated with an increased risk of fractures, loss of muscle mass and functional failure. In addition, the ever-increasing prevalence of these diseases is a major public health issue. There is a positive association between resistance/strengthening exercises and the prevention of osteoporosis and sarcopenia. In addition, protein and vitamin D supplements, as well as other vitamins and/or trace elements, seem to help in the better management of these diseases. However, due to the small number of samples available in most studies, it seems necessary to carry out randomized studies and meta-analyses of large population size and strictly defined criteria in order to draw valid conclusions on the effect of these interventions on osteoporosis and sarcopenia and to determine the length of time they should be applied in order to obtain long-term benefits in older people. There is also a need to further investigate the interaction between the bone tissue and the musculoskeletal system to enable the development of therapeutic regimens that target osteoporosis and sarcopenia simultaneously. In conclusion, the effects of exercise and nutrition on osteosarcopenia may suggest new prospects about the reduction of biomarkers which are secreted and act in both syndromes synergistically causing bone fractures and muscle degradation.

## Figures and Tables

**Table 1:** Studies investigating the effect of diet and/or exercise on the prevention, onset, and progression of osteoporosis.

Authors	Type of Article	Examined	Results
Muñoz-Garach et al., 2020 [37]	Review	Nutrition (vitamin D, calcium, trace elements, different types of food)	Eating a healthy diet contributes to bone health and reduces the risk of osteoporosis/fractures.
Malmir et al., 2020 [33]	Systematic review and meta-analysis	Nutrition (dairy products)	Conflicting results between cohort and case-control studies. The final conclusion was that dairy consumption does not reduce the risk of osteoporosis.
Pasqualini et al., 2019 [28]	Article	Resistance exercise	Resistance exercise enhances bones formation and performance on 1 RM.
Stanghelle et al., 2018 [46]	Randomized control trial	Exercise	A combination of resistance and balance exercises can help osteoporotic women.
Watson et al., 2018 [27]	Randomized control trial	High-intensity resistance and impact training (HiRIT)	HiRIT exercise was beneficial on BDM of post-menopausal women with osteoporosis and osteopenia.
Benedetti et al., 2018 [17]	Review	Exercise	Studies are still limited and no clear conclusions can be drawn. However, it seems that weight training and RT help osteoporotic people.
Isanejad et al., 2015 [47]	Article	Nutrition	Protein supplementation has a positive effect on body mass and prevention of osteoporosis, but more studies are needed.
Moreira et al. 2014 [29]	Article	Aerobic exercise	Aerobic exercise enhances bone formation and attenuates bone resorption.

**Table 2:** Studies investigating the effect of diet and/or exercise on the prevention, onset and progression of sarcopenia.

Authors	Type of Article	Examined	Results
Ganapathy and Nieves, 2020 [79]	Review	Nutrition (vitamin D, selenium, magnesium, calcium, etc.)	Especially vitamin D and proteins seem to have a protective role against sarcopenia and loss of muscle mass.
Moore et al., 2020 [56]	Umbrella review	Exercise	Little evidence for the effect of exercise on sarcopenia. Further studies needed.
Beaudart et al., 2019 [86]	Cohort study	Nutrition	Malnutrition is associated with an increased risk of sarcopenia.
Robinson et al., 2019 [39]	Review	Nutrition	The data are not yet sufficient to suggest a protective role of diet in sarcopenia.
Beckwée et al., 2019 [53]	Umbrella review	Exercise	Resistance exercises improve muscle mass, strength and physical activity.
Liao et al., 2019 [54]	Review	Exercise and diet	Strengthening exercises combined with protein supplements help to increase muscle mass and strength and improve mobility. When these supplements are combined with RT the positive effect is even greater for patients.
Sgrò et al., 2019 [77]	Review	Exercise and diet	The Mediterranean diet and protein/mineral supplements, as well as strengthening exercises, seem to have a positive effect on delaying the progression of sarcopenia.
Vlietstra and Hendrickx, 2018 [58]	Review	Exercise	The results are not yet clear, more studies with more rigorous and detailed inclusion criteria are needed.
Mohseni et al., 2017 [76]	Cross sectional	Nutrition	The evidence suggests that the Mediterranean diet contributes to the prevention of sarcopenia.
Steffl et al., 2017 [57]	Review and meta-analysis	Exercise	Physical activity such as aerobic exercise and strengthening exercises help prevent sarcopenia.
Yoshimura et al., 2017 [59]	Review and meta-analysis	Exercise and diet	Very limited evidence shows a positive association between exercise, diet and sarcopenia. More studies are needed.
Marzetti et al., 2017 [55]	Review	Exercise	Exercise helps to slow the progression of sarcopenia and increase muscle mass, but it is not yet known how long the duration of exercise has to be to produce long-term effects.
Wu, Pei-Yu, et al., 2020 [87]	Systematic review and meta-analysis	Exercise and diet	Both exercise and a combination of exercise and diet have beneficial effects on muscle strength and physical performance in older adults with sarcopenia
Bao et al., 2020 [88]	Systematic review and meta-analysis	Exercise	Exercise programs have the potential to support muscle function in older people with sarcopenia, which is recommended for daily life. Compared to muscle mass, muscle strength and physical performance can be improved to a greater extent with exercise.
Karlsson et al. 2020 [89]	Review	Nutrition	At an average age of 71 as a reflection of habitual eating habits, healthy eating patterns tend to protect against the development of sarcopenia over 16 years. In particular, increased adherence to a Mediterranean dietary pattern may be advantageous.
Granic et al., 2020 [90]	Systematic review	Nutrition	There was limited or inconclusive to moderate evidence for the role of food on muscle strength and sarcopenia in older adults. Although current dietary recommendations are often based on a nutrient approach, further research on the role of protein-rich and other foods in muscle health is needed.
Zhu et al., 2019 [91]	Article	Resistance exercise and nutrition	Resistance exercise program with and without nutrition supplementation improves strength factors.
Liao et al., 2017 [92]	Article	Resistance exercise	Resistance exercises attenuate muscle mass loss and prevent physical difficulty.

**Table 3:** Studies investigating the effect of diet and/or exercise on the prevention, onset and progression of both osteoporosis and sarcopenia (osteosarcopenia).

Authors	Type of Article	Examined	Results
Banitalebi et al., 2021 [97]	Article	Resistance exercise	Resistance exercise causes slight and insignificant improvement in osteoporosis markers.
Atlihan et al., 2020 [98]	Systematic review	Exercise	Increasing muscle mass and strength, but not in physical activity and bone transformation.
Fatima et al., 2019 [35]	Review	Exercise and diet (vitamin D)	Aerobic exercise does not affect muscle mass, unlike RT. Low levels of vitamin D are associated with an increased risk of osteoporosis and sarcopenia.
Huo et al., 2015 [98]	Cross-sectional study	Nutrition	Low intake of vitamins and amino acids are associated with the development of osteoporosis/sarcopenia in older people.

RT: Resistance Training.

## Data Availability

Not applicable.
